# Are Clinical Impairments Related to Kinematic Gait Variability in Children and Young Adults With Cerebral Palsy?

**DOI:** 10.3389/fnhum.2022.816088

**Published:** 2022-03-02

**Authors:** Anne Tabard-Fougère, Dionys Rutz, Annie Pouliot-Laforte, Geraldo De Coulon, Christopher J. Newman, Stéphane Armand, Jennifer Wegrzyk

**Affiliations:** ^1^Willy Taillard Laboratory of Kinesiology, Geneva University Hospitals and Geneva University, Geneva, Switzerland; ^2^Physical Therapy Unit, Department of Clinical Neurosciences, Lausanne University Hospital, Lausanne, Switzerland; ^3^Pediatric Orthopaedic Surgery Unit, Geneva University Hospitals, University of Geneva, Geneva, Switzerland; ^4^Pediatric Neurology and Neurorehabilitation Unit, Department of Pediatrics, Lausanne University Hospital, University of Lausanne, Lausanne, Switzerland; ^5^School of Health Sciences (HESAV), University of Applied Sciences and Arts Western Switzerland (HES-SO), Lausanne, Switzerland

**Keywords:** cerebral palsy, gait, variability, kinematic, clinical impairments

## Abstract

Intrinsic gait variability (GV), i.e., fluctuations in the regularity of gait patterns between repetitive cycles, is inherent to the sensorimotor system and influenced by factors such as age and pathology. Increased GV is associated with gait impairments in individuals with cerebral palsy (CP) and has been mainly studied based on spatiotemporal parameters. The present study aimed to describe kinematic GV in young people with CP and its associations with clinical impairments [i.e., passive range of motion (pROM), muscle weakness, reduced selective motor control (selectivity), and spasticity]. This retrospective study included 177 participants with CP (age range 5–25 years; Gross Motor Function Classification System I-III) representing 289 clinical gait analyses [*n* = 172 for unilateral CP (uCP) vs. 117 for bilateral CP (bCP)]. As variability metrics, Root Mean Square Deviation (RMSD) for nine lower-limb kinematic parameters and Gait Standard Deviation (GaitSD) – as composite score of the kinematic parameters – were computed for the affected (unilateral = uCP) and most affected side (bilateral = bCP), respectively, as defined by clinical scores. GaitSD was then computed for the non/less-affected side for between leg comparisons. Uni- and multivariate linear regressions were subsequently performed on GaitSD of the affected/most affected side with all clinical impairments (composite scores) as independent variables. Highest RMSD were found in the transverse plane (hip, pelvis), for distal joints in the sagittal plane (knee, ankle) and for foot progression. GaitSD was not different between uCP and bCP (affected/most affected side) but higher in the non-affected vs. affected side in uCP. GaitSD was associated with age (*p* < 0.001), gait deviation index (GDI) (*p* < 0.05), muscle weakness (*p* < 0.001), selectivity (*p* < 0.05), and pROM (*p* < 0.001). After adjustment for age and GDI, GaitSD remained associated with muscle weakness (uCP: *p* = 0.003, bCP: *p* < 0.001) and selectivity (bCP: *p* = 0.024). Kinematic GV can be expressed as global indicator of variability (GaitSD) in young people with CP given the strong correlation of RMSD for lower-limb kinematic parameters. In terms of asymmetry, increased variability of the non-affected vs. affected side may indicate contralateral compensation mechanisms in uCP. Notably muscle weakness (uCP, bCP) and selectivity (bCP) – but not spasticity – were associated with GaitSD. Further studies need to explore the clinical relevance of kinematic GV in CP to support the interpretation of clinical gait analyses and therapeutic decision-making.

## Introduction

Clinical Gait Analysis (CGA) is fundamental for the clinical management of pathological gait deviations. Intrinsic variability (i.e., cycle-to-cycle, within subject variability) occurring during one single testing session can be quantified using CGA. These temporal fluctuations in the regularity of gait patterns between repetitive cycles are inherent to the sensorimotor system and independent of error sources (extrinsic variability) (Schwartz et al., 2004). Intrinsic variability represents an important indicator of overall walking function (Stergiou et al., 2006) and can be a relevant parameter when interpreting CGA. Intrinsic variability depends on the neurological integration of multiple sensory inputs and the coordination of motor outputs (Wu et al., 2014; Pekny et al., 2015), and is influenced by factors such as age, walking speed, pathological and environmental conditions. In stable experimental environments (in which motor redundancy is reduced), low GV is usually considered as consistent and healthy, while increased GV is usually considered as less stable and pathological (Hausdorff, 2005). In contrast to simple variability measures (such as standard deviation and coefficient of variation) used to quantify data dispersion at specific instances of the gait cycle, more advanced variability metrics – such as the RMSD (Picerno et al., 2008) for unidimensional parameters, and GaitSD (Sangeux et al., 2016) as an overall index of kinematic GV – can characterize whole within-stride variability to quantify the similarity of curve patterns along the whole gait cycle (Di Marco et al., 2018). Association of these curve based metrics with clinical impairments could facilitate the interpretation of treatment efficacy on an individual basis.

Cerebral palsy (CP) represents a permanent, non-progressive “[…] disorder of movement and posture due to a defect or lesion of the immature brain” (Bax, 2008) and is the most frequent cause of motor disability in childhood (Pakula et al., 2009). Gait impairment in individuals with CP is complex and associated with clinical impairments such as spasticity, muscle weakness and reduced selective motor control. These sensorimotor deficits limit functional capacities within the locomotor system and may result in increased variability of kinematic and spatiotemporal parameters (Õunpuu et al., 2015). Heterogeneity in GV outcomes during the early stages of walking in young people with CP can also be explained by the degree of maturation associated with learning and neuroplasticity processes, rendering its interpretation challenging (Prosser et al., 2010).

In CGA, kinematic data are usually visualized as continuous data in the form of single-cycle curves for each joint, representing a time-varying value over one gait cycle (with stride-to-stride variability expressed as a set of curves over-plotted in the same graph). The mean curves of all gait cycles are visually inspected for each articulation/segment/plane and interpreted based on summary scores such as the gait deviation index (GDI) and the gait profile score (GPS) with regards to normative values of healthy control subjects. However, limited functional capacity (such as low walking speed) and compensation strategies of young people with CP impede a direct group comparison. Additionally, Oudenhoven et al. (2019) reported that even in typically developing (TD) children (especially at a young age), a substantial number of strides can be classified as abnormal with regards to stride-to-stride variability. Consequently, they concluded that a comparison of the mean curve of all gait cycles within one CGA session to that of an age-matched control group might lead to misinterpretation of gait deviations.

Given the particularly high intra-subject variability in neurodevelopmental disorders, mean values of discrete (spatiotemporal) parameters do not properly reflect individual gait characteristics (Sangeux et al., 2016). In contrast to spatiotemporal parameters, the variability of kinematic parameters remains largely unexplored in CP.

### Objectives

The aims of this study were to investigate kinematic GV in children and young adults with unilateral and bilateral spastic CP while (1) describing the pathology specific GV patterns of nine lower limbs kinematic variables and (2) identifying the explanatory variables of the variability pattern observed based on clinical impairment scores.

## Materials and Methods

### Participants

This retrospective study included young people with CP who underwent a CGA in the Kinesiology Laboratory of a tertiary hospital between 1994 and 2020. The local Ethics Committee approved this study (CER no. 2018-00229), i.e., permission to use and further process retrospective data recorded during CGA after anonymization. Informed consent was obtained from all participants and their respective legal guardians since this approval (March 2018). The local Ethics Committee granted a consent exemption for CGA performed prior to this date.

The inclusion criteria were: age between 5 and 25 years, diagnosis of unilateral or bilateral spastic CP (levels I to III on the Gross Motor Function Classification System-GMFCS) and the ability to walk 10 m without external support. The exclusion criteria were: lower limb surgery 12 months prior to the CGA, botulinum toxin injection (BTX) 6 months prior to the CGA, in case of multiple CGA time between each CGA < 1 year, and less than 5 valid kinematic gait cycles.

### Data Collection

Please refer to Table 1 for demographic and clinical participants’ characteristics.

**TABLE 1:** Differences of demographic, clinical and gait outcomes between participants with unilateral cerebral palsy (uCP, *n* = 105, 172 CGA) and bilateral cerebral palsy (bCP, *n* = 72, 117 CGA).

	uCP (172 CGA)	bCP (117 CGA)	Groups comparison		
			*P*	ES	95% CI
**Demographic and clinical characteristics**					
Age, years old	12.4 (4.8)	13.1 (5.1)	0.240	0.143	−1.9 to 0.5
Female, n (%)	79 (46%)	42 (36%)	0.115	0.146	−2 to 22%
Body weight status	–	–	0.361	–	–
Underweight, n (%)	18 (10%)	14 (12%)	0.835	0.003	−10 to 7%
Normal weight, n (%)	104 (60%)	80 (68%)	0.212	0.092	−20 to 4%
Overweight, n (%)	36 (21%)	19 (16%)	0.398	0.042	−5 to 14%
Obese, n (%)	12 (7%)	4 (3%)	0.300	0.063	−2 to 9%
GMFCS Level	–	–	<0.001*	–	–
I, n (%)	155 (90%)	73 (62%)	<0.001*	1.794	17–38%
II, n (%)	14 (8%)	37 (32%)	<0.001*	1.461	−34 to −13%
III, n (%)	0 (0.0%)	7 (6%)	0.004*	0.480	−11 to −1%
**Previous treatments**					
BTX > 6 months before, n (%)	72 (42%)	40 (34%)	0.234	0.083	−4 to 20%
Surgery > 1 year before, n (%)	62 (36%)	37 (32%)	0.515	0.025	−7 to 16%
**Composite impairment scores**					
Spasticity composite score, 0–16	1.1 (1.6)	3.0 (2.8)	<0.001*	0.902	1.4–2.5
Weakness composite score, 0–30	22.6 (4.0)	23.0 (5.1)	0.422	0.102	−1.6 to 0.7
Selectivity composite score, 0–12	9.5 (2.2)	9.4 (2.7)	0.751	0.039	−0.5 to 0.7
pROM composite score, 0–9	2.9 (1.7)	2.2 (1.7)	0.002*	0.387	0.3–1.0
**General gait characteristics**					
Walking speed, m/s	1.11 (0.15)	1.02 (0.25)	<0.001*	0.451	0.04–0.14
Normalized walking speed, (m/s)/LL	1.45 (0.30)	1.37 (0.39)	0.060	0.240	−0.01 to 0.17
Gait deviation index (GDI)	85.0 (11.3)	81.2 (12.1)	0.008*	0.331	1.1–6.6
**Gait asymmetry**					
Step time asymmetry, %	13.9 (7.7)	6.5 (6.6)	<0.001*	1.015	5.7–9.0
Step length asymmetry, %	8.7 (8.1)	8.3 (7.2)	0.665	0.051	−1.4 to 2.2
**Gait variability**					
GaitSD, degrees	2.5 (0.8)	2.5 (0.9)	0.970	0.005	−0.3 to 0.2
Step time CV, %	3.2 (1.9)	4.0 (2.3)	0.007*	0.365	−1.3 to −0.2
Step length CV, %	3.6 (2.1)	4.5 (3.1)	0.002*	0.432	−1.8 to −0.4

Statistical tests used were the student t-tests for continuous outcomes presented as mean [standard deviation (SD)] and Pearson chi-2 test for dichotomous outcomes presented as n (%). Significant differences between groups were considered at p < 0.05 (*).

*LL, leg length; CGA, clinical gait analysis; GDI, Gait deviation index; pROM, passive range of motion; GMFCS, gross motor function classification scale (Palisano et al., 2008); CV is coefficient of variation (SD/mean); BTX is botulinum toxin type A treatments; Spasticity composite score concerned the hip flexors, the knee flexors and extensors and the ankle plantar flexors; Weakness and selectivity composite score concerned the hip flexors and extensors, the knee flexors and extensors and the ankle plantar and dorsiflexors; pROM composite score concerned the hip extensors, the knee popliteal angle and the ankle dorsiflexors.*

To limit bias due to age and sex on the interpretation of body mass index (BMI) in a pediatric population, the BMI-for-age was computed as z-score (z) (de Onis et al., 2007) and weight status categories were defined using cut-offs recommended by the World Health Organization (de Onis and Lobstein, 2010).

#### Gait Analysis

Participants were instructed to walk barefoot at a comfortable self-selected speed along a 10 m walkway. Kinematic parameters were measured using a 12-camera motion analysis system (model Oqus 7+, Qualisys, Göteborg, Sweden) between 2015 and 2019, a 12-camera motion analysis system (Vicon MX3+, Vicon Peak, Oxford, United Kingdom) between 2007 and 2015 and a 6-camera motion analysis system (Vicon 460, Vicon Peak, Oxford, United Kingdom) before 2007. The marker trajectories were recorded at 100 Hz and filtered using the predicted mean-squared error filter MSE10 in the Nexus software before 2015 and high-pass 4th order Butterworth filter (10 Hz) after. Participants were equipped with 35 reflective markers placed on the skin at defined anatomical and technical landmarks according to the full-body Plug-in-Gait model (Davis et al., 1991).

#### Clinical Examination

The same day as CGA, an experienced physiotherapist clinically assessed the lower limbs including spasticity, selectivity, muscle weakness, and passive range of motion (pROM; Viehweger et al., 2007). In this retrospective study, several physiotherapists were involved in the clinical examination over time. Spasticity was evaluated using the Modified Ashworth scale (MAS), ranging from 0 the 4 where a 0 score is no spasticity (Bohannon and Smith, 1987). Selective motor control was evaluated using the selective control assessment of the lower extremity (SCALE) on a scale ranging from 0 to 2 where 0 is no selective control (Fowler et al., 2009). Muscle weakness was assessed by the manual muscle testing (MMT), ranging from 0 to 5 where 5 is no weakness (Hislop et al., 2014). The pROM was measured using a goniometer to the nearest 5°.

### Data Analysis

The affected and the most affected lower limb, respectively, were determined on the basis of higher clinical impairment composite scores (selectivity, spasticity, pROM, and muscle weakness, cf. section “Composite Clinical Score Analysis”) scaled from 0 to 1 each.

#### General Gait Parameters Analysis

The gait cycle was defined by the time between two foot strikes of the same foot and event detection was computed from the trajectory of markers placed on the pelvis and feet (Zeni et al., 2008) and checked manually. For each participant, five randomly selected gait cycles were included in the analysis. Walking speed (m/s), cadence (steps/min), step time (s), and step length (cm) were computed for all included gait cycles. In order to reduce bias related to the participants’ characteristics, both absolute and normalized (divided by leg length) walking speeds were reported (Hof, 1996).

The asymmetry of step time and step length were computed as abs[ln(left/right)] × 100% where 0% means perfect symmetry (Brændvik et al., 2020). The variability of step time and step length of the affected side/most affected lower-limb was computed with the coefficient of variation (CV) defined as the ratio between the standard deviation and the mean (calculated as SD/mean × 100%) of the included gait cycles (Brændvik et al., 2020).

#### Kinematic Variability Analysis

Lower limb kinematic parameters were computed according to a replication of the conventional gait model (Davis et al., 1991) using a custom-made software developed by Moveck^®1^. The quality of gait kinematic parameters was checked for artifacts and outliers based on the visual inspection of each curve.

A total of nine kinematic variables were used for the affected limb: pelvic tilt, pelvic obliquity, pelvic rotation, hip flexion, hip abduction, hip rotation, knee flexion, ankle dorsiflexion, and foot progression angle. For each of those kinematic parameters, the RMSD was computed based on five randomly included gait cycles (Picerno et al., 2008).

Additionally, the GaitSD was computed based on the five randomly included cycles for each limb to compare the variability of both legs. For all other analysis, GaitSD concerned the affected/most affected side. The GaitSD – a composite score of the kinematic variability – has previously shown satisfying (1) precision for a low number of gait cycles and (2) sensitivity to changes with age in typically developing children (Sangeux et al., 2016).

Finally, the GDI was computed based on the same five included cycles (Schwartz and Rozumalski, 2008).

#### Composite Clinical Score Analysis

Based on Papageorgiou et al. (2019), individual joint scores (hip, knee, and ankle) per impairment (spasticity, selectivity, weakness, and pROM) were calculated and then combined into a composite score for the affected/most affected limb.

More specifically, spasticity scores were: “ankle spasticity” (median scores of the gastrocnemius and soleus muscles, from 0 to 4), “knee spasticity” (sum of the knee flexor and extensor scores, from 0 to 8), and “hip spasticity” (scores of the hip flexors, from 0 to 4). The composite spasticity score was computed as the sum of all muscle groups, from 0 to 16.

Similarly, the composite weakness score (from 0 to 30) was computed as the sum of all weakness scores: “ankle weakness” (median score of the dorsiflexors with knee flexed and extended, summed up with ankle plantar flexors, from 0 to 10), “knee weakness” (sum of knee flexors and knee extensors scores, from 0 to 10), and “hip weakness” (sum of hip flexors and extensors scores, from 0 to 10).

The composite selectivity score was calculated as the sum of the joint selectivity scores according to the muscles associated via the SCALE (from 0 to 12).

Finally, the composite pROM score was calculated based on the “hip pROM” (Thomas test score, from 0 to 3), “knee pROM” (median of knee flexors and knee extensors, from 0 to 3), and “ankle pROM” (median score of triceps flexion and plantar flexion, from 0 to 3) with a total score from 0 to 9. All pROM scores were coded based on aged-gender normal dataset of Soucie et al. (2011) with code 0 for values inferior to 5th percentile (severe impairments), 1 for values between 5th and 50th percentile (moderate impairments); 2 for values between 50th and 75th percentile (low impairments), and 3 for values superior to 75th percentile (no or slight contractures).

#### Statistical Analysis

Statistical analysis was performed using R (version v.3.6.1) with the RStudio interface (version 1.2.5033). To describe demographic and clinical data, descriptive statistics were used and reported as the mean (SD = standard deviation) for continuous variables and as n (%) for dichotomous variables. Additionally, normality distribution of continuous outcomes was tested with the Shapiro–Wilk test. Differences between groups were tested with the Student *t*-test, differences between frequency distributions with the Chi-square test (χ^2^). Differences between groups were considered significant at *p* < 0.05.

Correlations between sets of the RMSD of all kinematic parameters were investigated using the R function “ggpairs” from the package GGally (version 1.5.0). Univariate linear regressions were performed on GaitSD with clinical composite scores as independent variables. Multivariate linear regressions were used to adjust for age and the gait deviation index (GDI). For each linear model, the linear regression coefficient β with the 95% confident interval (95% CI) and the adjusted R^2^ (Adj. R^2^) were reported. Adj. R^2^ was adjusted by the number of predictors in the model. Significant associations were considered at *p* < 0.05.

Finally, for young people with uCP, the GaitSD of the affected side was compared with the non-affected side using a paired Student *t*-test. Accordingly, the most affected side (side with the higher sum of scaled composite scores of clinical impairments) was compared with the less affected side in bCP.

## Results

### Participants

**FIGURE 1:**
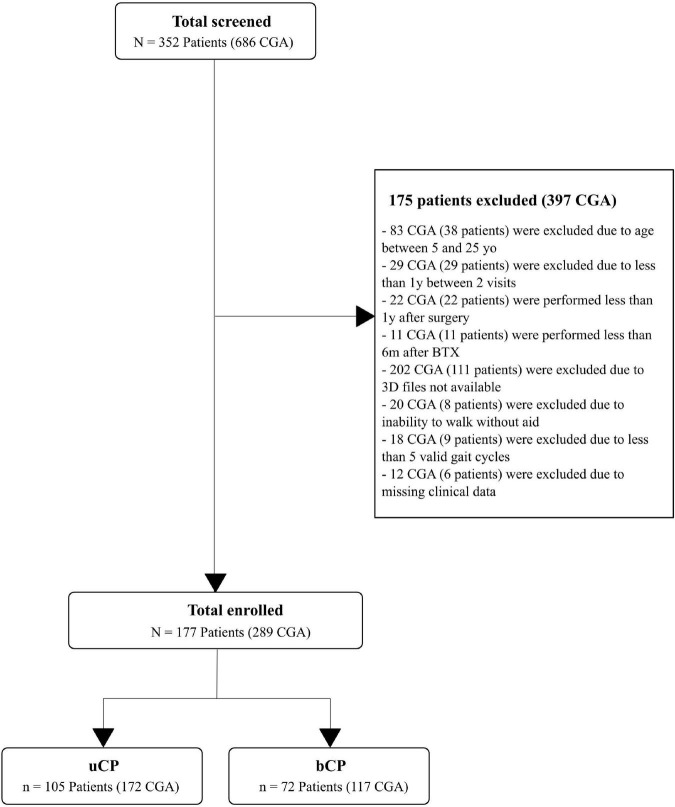
Flow chart of the inclusion and exclusion criteria leading to the selection of the 105 subjects with unilateral cerebral palsy (uCP) and 72 subjects with bilateral cerebral palsy (bCP). According to the inclusion and exclusion criteria, multiple visits were included resulting in a total of 172 clinical gait analysis (CGA) in the uCP and 117 CGA in the bCP group. 3D file available means gait records computed with the custom-made software developed by Moveck^®^ (https://moveck.com/). BTX is Botulinum toxin injection; yo, years old; y, year; m, months.

In total, 352 participants (686 CGA) with CP were screened: 41 (145 CGA) did not meet the following inclusion criteria: age between 5 and 25 years old, (>1 year between 2 CGA, >1 year after surgery, >6 m after BTX); 119 (222 CGA) did not have available 3D data (kinematics processed with Moveck) or walked with external aids; 9 (18 CGA) did not have > = 5 valid gait cycles and 6 (12 CGA) had missing clinical data (Figure 1). A total of 105 participants with uCP [172 CGA, 12.4 (4.8) years old, GMFCS: I (90%), II (10%)] and 72 participants with bCP [117 CGA, 13.1 (5.1) years old, GMFCS: I (62%), II (32%), III (6%)] were included (Figure 1).

As reported in Table 1, there were no significant differences between groups (uCP and bCP) for age, body weight status and previous treatments (BTX > 6 m before, Surgery > 1 year before), selectivity and weakness composite scores. They differed, however, with regard to GMFCS levels (*p* < 0.001), spasticity (*p* < 0.001, 95% CI: 1.4–2.5) and pROM (*p* < 0.001, 95% CI: 0.3–1.0) composite scores.

### Gait Characteristics

Compared to uCP, the bCP group had a significantly lower absolute walking speed (*p* < 0.001, 95% CI: 0.04–0.14), lower GDI (*p* = 0.008, 95% CI: 1.1–6.6), step time CV (*p* < 0.007, 95% CI: −1.3 to −0.2), and step length CV (*p* < 0.002, 95% CI: −1.8 to −0.4). In contrast, participants with bCP had a significantly lower step time asymmetry (*p* < 0.001, 95% CI: 5.7–9.0), but step length asymmetry was not significantly different between the groups (*p* = 0.665). Normalized walking speed, in contrast with absolute walking speed was not significantly different (*p* = 0.06) between uCP and bCP.

In uCP, step time was significantly higher (*p* < 0.001) on the affected side compared to the non-affected side [0.54 (0.07) vs. 0.47 (0.05) s] whereas step length was significantly reduced (*p* = 0.012) on the affected side compared to the non-affected side [0.55 (0.08) vs. 0.56 (0.08) m].

### Kinematic Variability

**FIGURE 2:**
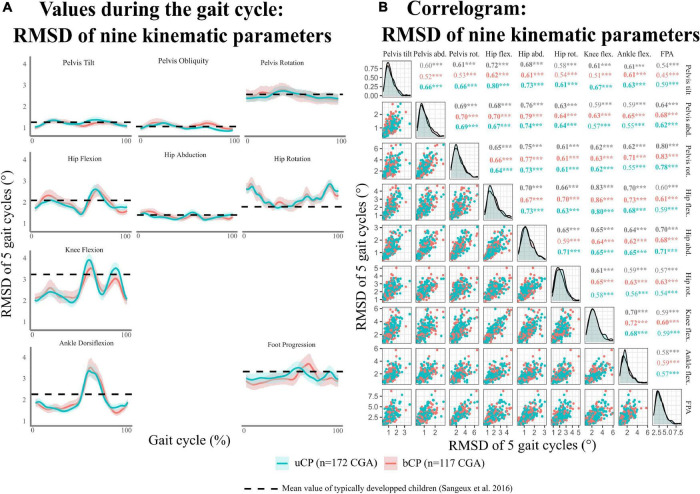
**(A)** Distribution of kinematic intrinsic variability [evaluated using root mean square deviation (RMSD) for five gait cycles of the same visit] during gait cycle per kinematic parameters. **(B)** Correlogram of the mean RMSD for kinematic parameters. uCP, participants with unilateral cerebral palsy; bCP, participants with bilateral cerebral palsy; CGA, clinical gait analysis. Level of significance of Pearson correlations *p < 0.05; **p < 0.01; ***p < 0.001.

As reported in Figure 2A, the highest values of RMSD (>2°) were found in the transverse plane and distal joints (knee, ankle). The RMSD of all nine kinematic variables were significantly correlated (*r* > 0.50, *p* < 0.05) (Figure 2B).

**FIGURE 3:**
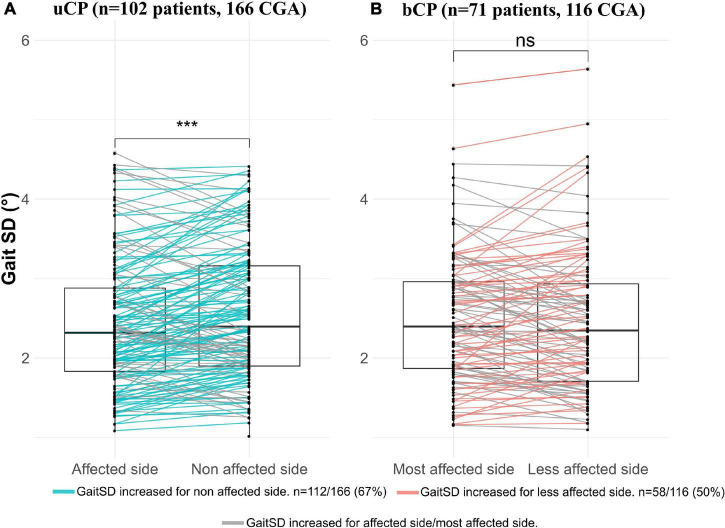
Comparison of gait standard deviation (GaitSD) between: **(A)** Affected vs. non-affected side in participants with unilateral cerebral palsy (uCP); **(B)** Most affected side vs. less affected side in participants with bilateral cerebral palsy (bCP). Due to absence of minimum five valid gait cycles in both sides, six CGA (three patients) were excluded in uCP and one CGA (one patient) in bCP. Level of significance of paired Student *t*-tests *p < 0.05; **p < 0.01; ***p < 0.001.

Gait Standard Deviation was not significantly different between the uCP and bCP groups (Table 1) and it was significantly higher on the non-affected side compared to the affected side in uCP (Figure 3A). On the contrary, GaitSD was not significantly higher in the most affected lower-limb compared to the less affected limb in bCP (Figure 3B). GaitSD was increased in the non-affected side compared to the affected side in 67% of all CGA in the uCP group (112 out of 166 CGA). This proportion was significantly higher (*p* = 0.005) than that of CGA performed by participants with bCP for which no increased GaitSD could be observed in the less affected side compared to the most affected side (50%; 58 out of 116 CGA).

### Relation Between Age, Gait Deviation Index, Composite Impairment Scores and Gait Standard Deviation

**FIGURE 4:**
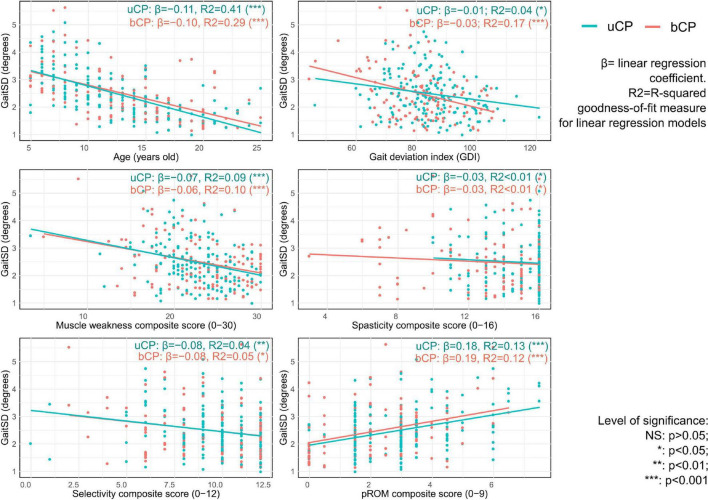
Univariable linear regression results with scatterplot of gait standard deviation (GaitSD in degrees) for age, gait deviation index and composite scores of clinical impairments [spasticity, selectivity, muscle weakness, and passive range of motion (pROM)] of 105 subjects with unilateral cerebral palsy [uCP, 172 clinical gait analysis (CGA)] and 72 subjects with bilateral cerebral palsy (bCP, 117 CGA).

As reported in Figure 4, univariate linear regression showed that GaitSD was significantly associated with age (uCP: *R*^2^ = 0.41, *p* < 0.001, bCP: *R*^2^ = 0.29, *p* < 0.001), GDI (uCP: *R*^2^ = 0.04, *p* = 0.012, bCP: *R*^2^ = 0.17, *p* < 0.001), muscle weakness (uCP: *R*^2^ = 0.09, *p* < 0.001, bCP: *R*^2^ = 0.10, *p* < 0.001), selectivity (uCP: *R*^2^ = 0.04, *p* = 0.007, bCP: *R*^2^ = 0.05, *p* = 0.021), and pROM (uCP: *R*^2^ = 0.13, *p* < 0.001, bCP: *R*^2^ = 0.12, *p* < 0.001). As reported in Table 2, only muscle weakness (uCP: *R*^2^ = 0.443, *p* = 0.011, bCP: *R*^2^ = 0.387, *p* = 0.009) and selectivity (bCP: *R*^2^ = 0.361, *p* = 0.024) remained significantly correlated with GaitSD after adjustment for age and GDI.

**TABLE 2:** Multiple-linear regression analysis of cycle-to-cycle gait kinematic variability (GaitSD) and clinical composite scores [weakness, spasticity, selectivity, and passive range of motion (pROM)] in subjects with unilateral cerebral palsy (uCP, *n* = 105, 172 CGA) and bilateral cerebral palsy (bCP, *n* = 72, 117 CGA).

	uCP (*n* = 172 CGA)			bCP (*n* = 117 CGA)		
Outcome	β (95% CI)	*p*-value	Adj. R^2^	β (95% CI)	*p*-value	Adj. R^2^
**Weakness composite score**						
Unadjusted	−0.06 (−0.10, −0.03)	<0.001*	0.086	−0.06 (−0.09, −0.02)	<0.001*	0.092
Age adjusted	−0.04 (−0.06, −0.01)	0.003*	0.433	−0.06 (−0.08, −0.03)	<0.001*	0.371
GDI adjusted	−0.06 (−0.09, −0.03)	<0.001*	0.098	−0.03 (−0.07, 0.01)	0.061	0.139
Age + GDI adjusted	−0.03 (−0.06, −0.01)	0.011*	0.443	−0.04 (−0.07, −0.01)	0.009*	0.387
**Spasticity composite score**						
Unadjusted	−0.03 (−0.11, 0.05)	0.489	0.003	−0.03 (−0.09, 0.03)	0.353	0.001
Age adjusted	−0.05 (−0.11, 0.01)	0.127	0.411	−0.05 (−0.10, 0.01)	0.077	0.297
GDI adjusted	−0.02 (−0.10, 0.06)	0.558	0.030	0.01 (−0.05, 0.07)	0.726	0.111
Age + GDI adjusted	−0.04 (−0.11, 0.02)	0.154	0.428	−0.02 (−0.07, 0.04)	0.534	0.350
**Selectivity composite score**						
Unadjusted	−0.08 (−0.14, −0.02)	0.007*	0.037	−0.08 (−0.14, −0.01)	0.021*	0.039
Age adjusted	−0.04 (−0.09, 0.01)	0.057	0.416	−0.08 (−0.14, −0.03)	0.036*	0.330
GDI adjusted	−0.07 (−0.13, −0.01)	0.017*	0.061	−0.03 (−0.09, 0.04)	0.469	0.115
Age + GDI adjusted	−0.04 (−0.08, 0.01)	0.109	0.430	−0.05 (−0.10, −0.01)	0.024*	0.361
**pROM composite score**						
Unadjusted	0.19 (0.12, 0.26)	<0.001*	0.134	0.19 (0.10, 0.029)	<0.001*	0.112
Age adjusted	0.07 (−0.04, 0.13)	0.065	0.415	0.05 (−0.06, 0.15)	0.384	0.281
GDI adjusted	0.19 (0.12, 0.26)	<0.001*	0.170	0.22 (0.14, 0.32)	<0.001*	0.269
Age + GDI adjusted	0.06 (−0.01, 0.12)	0.055	0.434	0.10 (−0.01, 0.20)	0.058	0.369

Univariate and multivariate linear regression were used. β is the linear regression coefficient; 95% CI is the 95 confident interval; R^2^ is the proportion of the variance accounted for the dependent variable. Adj. R^2^ has been adjusted for the number of predictors in the model. Significant associations were considered at p < 0.05 (*).

*CGA is clinical gait analysis; pROM is passive range of motion; GDI is Gait Deviation index; Spasticity composite score concerned the hip flexors, the knee flexors and extensors and the ankle plantar flexors; Weakness and selectivity composite score concerned the hip flexors and extensors, the knee flexors and extensors and the ankle plantar and dorsiflexors; pROM composite score concerned the hip extensors, the knee popliteal angle and the ankle dorsiflexors.*

## Discussion

The aim of this study was to investigate kinematic GV in children and young adults with unilateral and bilateral spastic CP while (1) describing pathology specific GV patterns of nine lower-limb kinematic variables and (2) identifying the explanatory variables of the variability pattern observed based on clinical impairment (composite scores). Results are interpreted separately for uCP and bCP as evidence suggests that lower-limb motor functioning differs based on the topographical classification of CP (Meyns et al., 2016).

For both groups (uCP and bCP), kinematic variability was highest for distal joints (knee, ankle) in the sagittal plane, for proximal joints (hip and pelvis) in the transverse plane, and for the foot progression angle. These results agree with those of Sangeux et al. (2016) showing increased stride to stride variability (GV SD > 3) for identical planes and joints in TD children of similar age (6–17 years). Consequently, kinematic variability characteristics per joint location and planes observed in participants with uCP and bCP do not seem to be related to pathological variability. Future studies including a matched control group would need to confirm this finding.

Moreover, the present results show no joint/segment specific kinematic GV pattern in young people with CP highlighted by the correlation of all nine RMSD parameters of the affected/most-affected limb in uCP and bCP. Consequently, the use of the GaitSD as a composite score – sensitive to changes with age and suggested for TD children (Sangeux et al., 2016) – can be applied in youngsters with CP. The inclusion of the GaitSD, allowing for intra-individual evaluation in clinical interpretation of CGA, might complement gait deviation scores [such as the gait profile score (GPS) and GDI] that refer to a norm (inter-individual comparison). As recently shown, even young TD children show high stride-to-stride variability with many strides classified as “abnormal” when compared to group averaged normalized curves (Oudenhoven et al., 2019). Thus, an interpretation of CGA based on the comparison of the mean curve of repetitive cycles within one session to normative values ignores both physiological and pathology-related intra-individual variability that can be a relevant clinical information for the understanding of gait deviations.

Another result of the present study is that the GaitSD and RMSD outcomes of the affected/most affected side were not significantly different between participants with uCP and bCP. This finding was contrary to our hypothesis that kinematic variability would be increased on the most affected side in bCP compared to the affected side in uCP due to more severe clinical impairments. Indeed, we observed higher levels of spasticity, pROM, (overall) GFMCS and lower GDI in bCP compared to uCP, in agreement with previous research stating higher clinical impairments in bCP (Prosser et al., 2010; Meyns et al., 2016) even if the between group difference in GDI was not clinically significant (<10°). In previous literature, the comparison between variability characteristics in individuals with uCP vs. bCP has only been performed on spatiotemporal parameters without considering the implication of each lower limb. In agreement with Brændvik et al. (2020), we observed significantly higher step time variability and step length variability in bCP compared to uCP suggesting that adjustability of foot placement and timing is less affected in uCP.

Consequently, we investigated asymmetry characteristics of kinematic variability by comparing the affected/more affected side with the non/less affected side in both groups. Interestingly, GaitSD was increased on the non-affected side compared to the affected side in uCP. No inter-limb asymmetry based on the GaitSD comparison was found in bCP. Higher variability in the non-affected limb might be associated with the significantly shorter step time and higher step length observed in the non-affected vs. the affected limb in our population with uCP. Performing a longer distance during a shorter amount of time might suggest less refined motor skills inducing higher kinematic variability, however, this hypothesis requires further investigation. Alternatively, increased variability in the non-affected limb might reflect compensation via non-damaged cortical areas leading to higher motor capacity (through a broader motor repertoire allowing adjustment to motor deficits of the affected side). Such “good” variability might be associated with increased connectivity of the non-affected side as shown for stroke patients (Bajaj et al., 2015) and could explain the increased overall functional capacity in young people with uCP compared to bCP. Future studies would need to confirm the presence and origin of contralateral compensation patterns through increased variability and investigate underlying mechanisms. In terms of coordination of neural control across legs, Bulea et al. (2017) reported preserved control circuits in children with uCP allowing each leg to adapt independently to reduce gait asymmetry in response to external perturbations. This finding complements our finding of variability and asymmetries in the uCP group. Common research and practice that currently focuses on the affected side in uCP (Sangeux et al., 2013) should therefore not neglect the influence of the unaffected leg on gait patterns.

Concerning the second objective of this study, GaitSD was significantly associated with age, GDI and all composite scores (except spasticity) in both groups. The absence of association between GaitSD and the spasticity composite score could be explained by the relatively low level of spasticity in our sample compared to a previously investigated population (Papageorgiou et al., 2019).

The significant association between GaitSD and age is in line with the results reported by Sangeux et al. (2016) and Oudenhoven et al. (2019) in TD children showing high gait variability at a young age. The impact of gait maturity on kinematic GV should be considered in the clinical context to better support the interpretation of gait deviations in children with CP. To our knowledge, our study is the first to report a significant association between GaitSD and GDI in young people with uCP and bCP. Even though overall GaitSD level was similar to values reported in TD children, GaitSD was increased in participants with higher gait deviations, especially in bCP.

Concerning clinical composite scores, GaitSD remained significantly associated with muscle weakness in both groups after adjustment for age and GDI. These results are in line with Chang et al. (2013) who reported for patients with chronic stroke that weakness (rather than spasticity) affects voluntary force control and leads to higher force variability in isometric muscle contractions. However, even though muscle strength has been reported to be correlated with gait function in CP (Desloovere et al., 2006; Ross and Engsberg, 2007), the association between static isometric muscle contractions and complex gait is not straightforward in individuals with CP (Dallmeijer et al., 2011) and requires further investigation. Moreover, increased GaitSD was associated with reduced selective motor control after correction for age and GDI in bCP. In individuals with CP, the motor cortex and/or tracts are damaged which may affect volitional control of movement (Fowler, 2010) and spinal mechanisms responsible for the automatic control of gait (Clowry, 2007). Chruscikowski et al. (2017) observed a negative correlation between selectivity and the gait profile score (as an indicator of gait abnormality) in young people with bCP and hypothesized less complex control strategies during gait as the underlying neurological cause (Chruscikowski et al., 2017).

The present study has several limitations. First, the included participants with CP globally had mild to moderate clinical impairments. Our findings would need to be confirmed in young people with CP with more severe clinical impairments. Second, low to moderate inter-operator reliability of clinical evaluation (spasticity, selectivity, muscle weakness, and pROM) (Fosang et al., 2003) could have influenced the results with regards to the second objective of the present study. Another limitation is the fact that data acquisition (including technology) and processing have evolved over the years and unfortunately quality assurance was not uniform/standardized the same way over time. Finally, the calculation of the GaitSD is only based on five randomly selected cycles. According to Sangeux et al. (2016), a minimum of six strides is recommended for healthy subjects and up to 10 strides in pathological populations due to increased GV baseline values. In practice, however, 10 strides considerably increase the number of walking trials having the risk of inducing fatigue in populations with neuromotor disorders.

The presented metrics evaluating kinematic GV (i.e., RMSD and GaitSD) detect variations in amplitude and shape of the curve and are sensitive to time-shift between the curves (Di Marco et al., 2018). Temporal alignment could be achieved using existing techniques such as dynamic time warping (Helwig et al., 2011). Future studies could additionally investigate non-linear measures to assess gait variability (such as Largest Lyapunov Exponent), and gait regularity (such as approximate Entropy) (Stergiou et al., 2004).

Finally, gait at a self-selected speed in laboratory conditions does not reflect the complexity of gait in daily life. Investigations on the impact of cognitive load, walking surface and gait speed variations, would complement our findings of GV in individuals with CP.

## Conclusion

Kinematic GV can be expressed as global indicator (GaitSD) in children and young adults with CP due to the strong correlation of the RMSD for the investigated lower-limb kinematic parameters. Increased variability on the non-affected side suggests contralateral compensation patterns in participants with uCP. After correction for age and GDI, GaitSD remained significantly associated with pathology-specific muscle weakness for the uCP and bCP groups and selectivity for the bCP group. Further studies need to explore the clinical relevance of kinematic GV in individuals with CP to support the interpretation of clinical gait analysis and therapeutic decision-making.

## Data Availability Statement

The data analyzed in this study is subject to the following licenses/restrictions: clinical data. Requests to access these datasets should be directed to the corresponding author.

## Ethics Statement

The studies involving human participants were reviewed and approved by the Regional Research Ethics Committee (CCER) Geneva. Written informed consent to participate in this study was provided by the participants’ legal guardian/next of kin.

## Author Contributions

AT-F, DR, AP-L, GD, CN, SA, and JW made substantial contributions to the conception or design of the work, the acquisition, analysis, or interpretation of data for the work, drafting the work or critically revising it for important intellectual content, and final approval of the version to be submitted for publication. All authors further agreed to be accountable for all aspects of the work in ensuring that questions related to the accuracy or integrity of any part of the work are appropriately investigated and resolved.

## Conflict of Interest

The authors declare that the research was conducted in the absence of any commercial or financial relationships that could be construed as a potential conflict of interest.

## Publisher’s Note

All claims expressed in this article are solely those of the authors and do not necessarily represent those of their affiliated organizations, or those of the publisher, the editors and the reviewers. Any product that may be evaluated in this article, or claim that may be made by its manufacturer, is not guaranteed or endorsed by the publisher.

## Footnotes

1
https://moveck.com/
